# Video-based diagnosis support system for pianists with Musician’s dystonia

**DOI:** 10.3389/fneur.2024.1409962

**Published:** 2024-07-02

**Authors:** Takanori Oku, Shinichi Furuya, André Lee, Eckart Altenmüller

**Affiliations:** ^1^College of Engineering and Design, Shibaura Institute of Technology, Tokyo, Japan; ^2^Sony Computer Science Laboratories, Inc., Tokyo, Japan; ^3^NeuroPiano Institute, Kyoto, Japan; ^4^Institute of Music Physiology and Musicians’ Medicine, University of Music, Drama and Media, Hanover, Germany; ^5^Department of Neurology, Klinikum rechts der Isar, Technical University of Munich, München, Germany

**Keywords:** Musician’s dystonia, pianists, machine learning, video-based diagnostic method, hand biomechanics

## Abstract

**Background:**

Musician’s dystonia is a task-specific movement disorder that deteriorates fine motor control of skilled movements in musical performance. Although this disorder threatens professional careers, its diagnosis is challenging for clinicians who have no specialized knowledge of musical performance.

**Objectives:**

To support diagnostic evaluation, the present study proposes a novel approach using a machine learning-based algorithm to identify the symptomatic movements of Musician’s dystonia.

**Methods:**

We propose an algorithm that identifies the dystonic movements using the anomaly detection method with an autoencoder trained with the hand kinematics of healthy pianists. A unique feature of the algorithm is that it requires only the video image of the hand, which can be derived by a commercially available camera. We also measured the hand biomechanical functions to assess the contribution of peripheral factors and improve the identification of dystonic symptoms.

**Results:**

The proposed algorithm successfully identified Musician’s dystonia with an accuracy and specificity of 90% based only on video footages of the hands. In addition, we identified the degradation of biomechanical functions involved in controlling multiple fingers, which is not specific to musical performance. By contrast, there were no dystonia-specific malfunctions of hand biomechanics, including the strength and agility of individual digits.

**Conclusion:**

These findings demonstrate the effectiveness of the present technique in aiding in the accurate diagnosis of Musician’s dystonia.

## Introduction

Musician’s dystonia (MD) is a task-specific movement disorder that leads to deterioration or loss of control of highly skilled movements at the musical instrument. This career-threatening disorder has a life prevalence of about 1–2% amongst professional musicians (1) and is thus considerably more frequent as compared to writer’s cramps, another form of task-specific focal dystonia, with a prevalence of about 0.08 (2). In the beginning, typical symptoms are subtle: feelings of tension in the forearm muscles when playing, slowing down of fast repetitive movements, and irregularity of movements required to be regular, e.g., scale playing in classical music. Later, curling of fingers or uncontrolled extension whilst playing, “sticking” fingers at the keys due to overactivity of finger-flexor muscles, and visible cramping of wrist extensors and flexors are more obvious (3), and can be observed objectively and assessed via video-rating of informed and trained raters (4).

Risk factors for Musician’s dystonia include genetic predisposition (5), workload at the instrument (1, 6), and over-practice through highly repetitive movements over a long period (7). The diagnosis of the MD is clinical and not straightforward since it requires examination at the instrument and thus, musical knowledge and knowledge of the playing technique of the examiner. For example, the first symptoms usually include unevenness of regular scale-playing, a symptom which develops insidiously and is perceived by musicians; however, it is frequently not perceived by medical experts who have no musically trained ear. Therefore, there is an urgent need to develop a method for diagnosing Musician’s dystonia that can be easily used even by those medical doctors with no expertise in music and musical performance.

The purpose of this study was to develop a “Red Flag” system of biological markers by objectively assessing playing skill at the piano to facilitate medical personnel’s diagnosis of Musician’s dystonia. This diagnosis system draws on artificial intelligence and trained machine learning algorithms. Meanwhile, many diagnostic methods using artificial intelligence (AI) technology are being developed. The application of AI technology in medical imaging diagnosis, for example, is very promising, and there are areas where it surpasses the performance of specialists (8, 9). However, the diagnosis of movement disorders has not progressed as far as medical imaging diagnosis.

In this study, we present a video-based Musician’s dystonia diagnostic system targeting pianists. Previous studies have shown that pianists with focal dystonia have impaired coordination of finger movements (10). Based on this, we use a kinematics-based approach supplemented by video recordings. In addition, we undertook research into the task specificity of Musician’s dystonia and into possible biomechanical risk factors (11). Aberrant anatomical connections between tendons and muscles for example may increase stiffness and the load on specific, mostly extrinsic and intrinsic finger flexor and extensor muscles, which in turn can trigger focal dystonia (12). Indeed, abnormalities in biomechanical conditions impacting finger joint movements were reported to be associated with focal hand dystonia (10, 12), indicating that not only neurological factors but also anatomical and biomechanical factors could be involved. Therefore, we also examined differences in biomechanical functions that are not directly relevant to the piano performance in pianists suffering from Musician’s dystonia and in healthy pianists.

## Methods

### Participants and tasks

Participants were 20 healthy pianists (25.2 ± 4.1 years old, 30% male) and 16 pianists (47.0 ± 10.9 years old, 56.2% male) suffering from focal hand dystonia. All participants were professional pianists with a master’s degree in piano performance. 19 of the healthy and 16 of the dystonic pianists were reported to be right-handed individuals.

Musicians with dystonia were diagnosed by the senior author of the paper (EA), an internationally acknowledged movement disorders specialist and neurologist. The affected hands and fingers varied. Unfortunately, due to the rarity of the disorder, it was not possible to match the gender and age to those of the control group. Data on age, gender, duration of being affected by dystonia, affected hand, and handedness are summarized in Table 1, while data on age, gender, handedness, and piano playing experience of participants in the healthy control group are summarized in Table 2.

**Table 1:** Characteristics of pianists with Musician’s dystonia.

	Age	Gender	Affected hand	Handedness	Duration of being affected (year)	Dystonic pattern
A	57	F	R	R	9	Index and middle finger flexion
B	26	F	R	R	2	Ring and little finger flexion
C	34	M	R	R	2	Middle finger and wrist flexion
D	59	M	R	R	16	Thumb and index finger flexion
E	42	M	R	R	1	Thumb and index finger flexion
F	46	M	R	R	22	Index finger flexion
G	43	F	L	R	5	Ring finger flexion
H	36	M	R	R	3	Index and middle finger flexion
I	56	M	R	R	40	Middle, ring and little finger flexion
J	53	M	R	R	26	Middle finger flexion
K	51	F	R	R	30	Middle finger flexion
L	53	F	R	R	11	Middle and ring finger flexion
M	29	F	R	R	10	Index and middle finger flexion
N	47	M	R	R	17	Index and middle finger flexion
O	64	M	L	R	14	Wrist flexion
P	57	F	R	R	27	Middle and ring finger flexion
	47.0 ± 10.9	F:M = 7:9	R:L = 14:2	R:L = 16:0	14.7 ± 11.6	

**Table 2:** Characteristics of healthy control participants.

	Age	Gender	Handedness	Years of playing the piano
A	25	M	R	12
B	24	M	R	19
C	21	F	R	15
D	23	F	R	18
E	31	F	R	28
F	20	F	R	16
G	22	F	R	18
H	21	F	R	18
I	24	F	R	21
J	38	F	R	32
K	25	F	L	20
L	20	M	R	16
M	28	F	R	23
N	28	F	R	23
O	28	M	R	22
P	25	F	R	21
Q	29	F	R	24
R	23	M	R	18
S	22	F	R	18
T	24	M	R	19
	25.2 ± 4.06	F:M = 14:6	R:L = 19:1	20.1 ± 2.18

All participants gave informed consent, and the Ethics Committee of Leibniz University Hannover approved the study.

### Piano performance measurements

**Figure 1:**
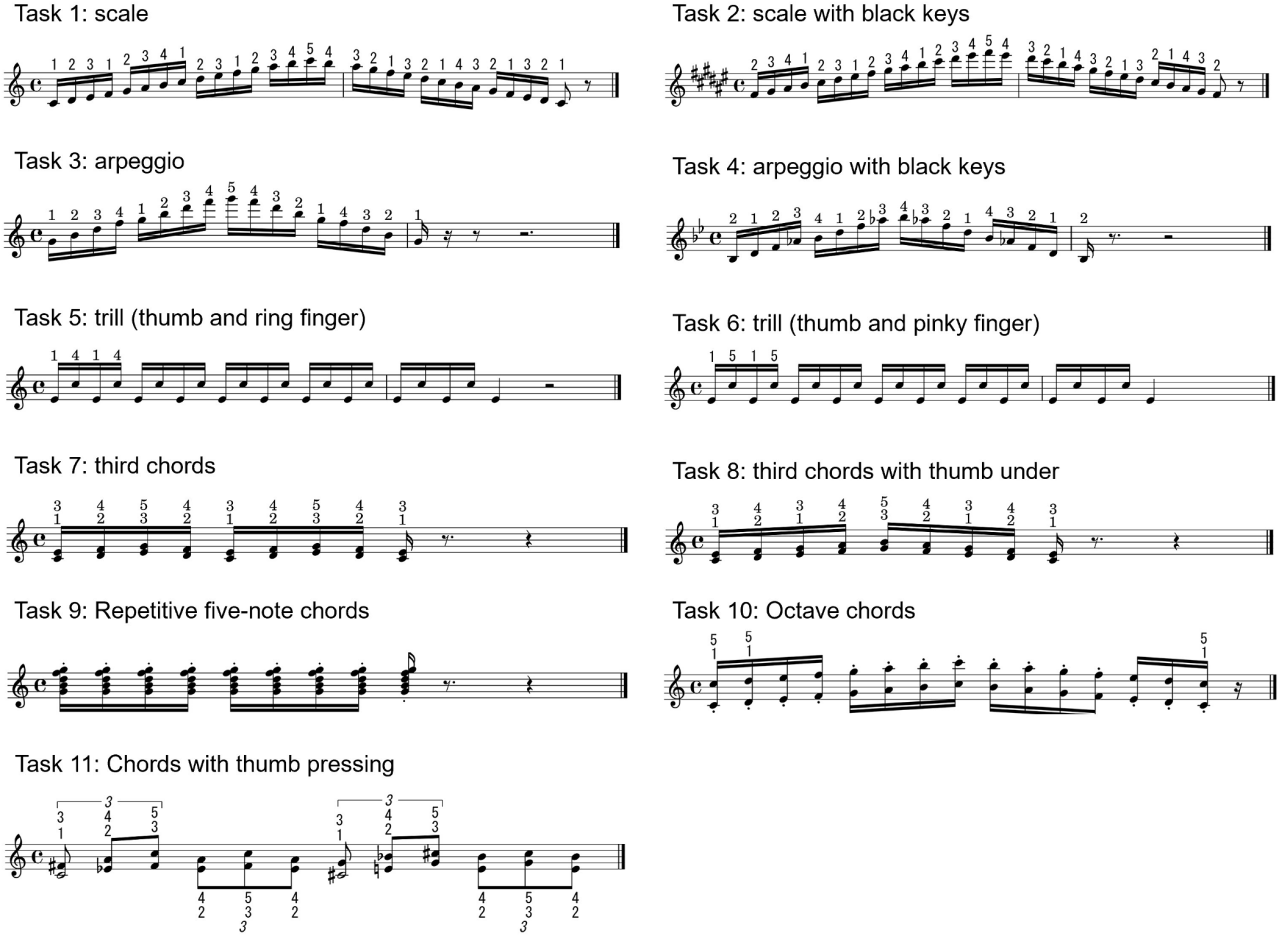
Eleven pianistic tasks participants performed. The pianistic tasks consist of sequence of monophonic notes such as scale, arpeggio, and trill, and chord strikings such as third chords, repetitive chord, and octave.

Participants performed 11 pianistic tasks on a Steinway B-Grand piano. These pianistic tasks included single-note sequences such as scales, arpeggios, and trills and chord-striking sequences such as thirds, repetitive five-note chords, and octaves (Figure 1). These movement sequences are frequently used in piano performance, and some of these are also used in the diagnosis of dystonia (13). Participants were instructed to perform with designated fingering. Furthermore, each exercise was performed at two loudness levels (pp and ff) and two tempi, a fixed tempo and the fastest possible tempo for each individual participant. The fixed tempo was set at 100 bpm for single-note sequences (tasks 1–6) and 80 bpm for chord-striking sequences (tasks 7–11). All performances were played bimanually with both hands, using mirror-image fingering.

**Figure 2:**
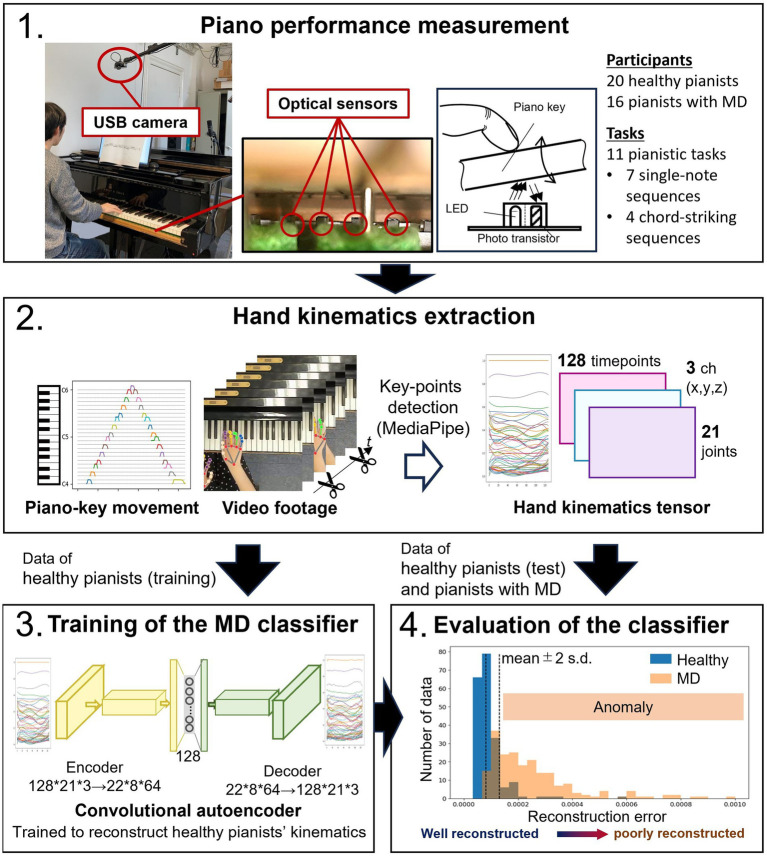
Schematic illustration of the video-based diagnostic support system. The video footage was recorded using a commercially available USB camera, and the movement of the piano keys was captured by a custom-built contactless measurement system using optical sensors (14). Hand kinematics during piano performance were measured using a video-based motion capture system (MediaPipe). The kinematic data of healthy pianists were used to train a convolutional autoencoder. This convolutional autoencoder trained with healthy pianists’ data effectively reconstructs healthy pianists’ hand kinematics. However, it cannot accurately reconstruct the data with different kinematic features, i.e., the data of pianists with MD.

In order to obtain as naturalistic tactile and proprioceptive information from the action of the grand piano as possible, a custom-made high-precision measurement system (14) capable of non-contact measurement inside a grand piano was installed. This measurement system records the movements of all the 88 piano keys with 1 millisecond temporal resolution and 0.01 mm spatial resolution. A USB commercial camera was mounted 1.0 m above the piano keyboard so that the C4 key is at the center of the field of view. The USB camera recorded the video footage of hand and finger movements during the performance with a 78-degree angle of view and a resolution of 1280*720 at 60 Hz (BRIO, Logicool co.). The field of view was 1620*911 mm at the keyboard surface, with one pixel corresponding to approximately 1.3 mm. Since the width of the white keys is approximately 22.5 mm, the camera has a resolution of 17 segments for each key, providing an enough high resolution to measure hand motions. Video-footage from a side camera were often used at video-based diagnosis of MD in previous studies. However, because of the difficulty of controlling the focus when the distance from camera to hand significantly changed during playing, we did not include the video-footage from side cameras into the algorithm. Kinematics were measured using the hand landmark detection of the MediaPipe Python library (15). MediaPipe is a framework for building machine learning pipelines for processing time-series data such as video and audio. Using MediaPipe makes it possible to detect the positions of 21 anatomical landmarks of the hand, including the joint positions of each finger and the positions of the fingertips and wrists, in three-dimensional coordinates on each video frame. Movement of piano keys and video footage are synchronously recorded using an internal clock of the measurement system. A display of the set-up is shown in Figure 2-1.

### Machine learning algorithm

For the video-based diagnostic support system, we developed an anomaly detection system using an autoencoder. An autoencoder is a type of artificial neural network structure that is trained to reconstruct input data so that the input and output data are identical (16). It has an hourglass-like structure with a middle layer of fewer dimensions than the layers representing input/output data. The network structure for extracting features that represent the input data is trained from the input layer to the middle layer (encoder), and a network structure for expanding from the extracted features to the original data is trained from the middle layer to the output layer (decoder). Therefore, when a trained autoencoder encounters unfamiliar data with features different from the training data sets, the reconstruction accuracy decreases since it cannot reconstruct features that are not included in the training data sets well. This characteristic makes autoencoders useful in anomaly detection (17). In this study, we trained the autoencoder using the kinematic data of healthy pianists during piano performance as training data. If the kinematics of healthy pianists and pianists with MD have different features, we can discriminate between the performances of MD patients and healthy pianists using the reconstruction accuracy.

We used the time series data of the coordinates of the hand and finger landmark points output by MediaPipe as input data for the autoencoder. We extracted the video frames corresponding to the beginning of the first keystroke to the end of the last keystroke based on the vertical displacement data of the keys. Using MediaPipe’s hand detection for each extracted frame, we obtained the time-series data of the positions of the 21 feature points of the hand in three dimensions during the performance. Each performance data was time normalized by adjusting the number of data points to 128 through spline interpolation. As a result, the kinematic data of each performance is shaped into a tensor of 21 × 3 × 128. Since pianists with focal dystonia showed abnormal spatiotemporal coordination between adjacent fingers/joints (10), we used a convolutional autoencoder that can compress information while preserving adjacent spatiotemporal information. The autoencoder consisted of 7 layers of convolutional encoders and 7 layers of convolutional decoders. We performed data augmentation to make the estimation results robust against different camera mounting conditions. Here, we used rotation of the coordinates extracted by MediaPipe, scaling in the vertical and horizontal directions, and their combination. This augmentation was done to simulate possible camera mounting deviations. We randomly divided the healthy participants’ data into 80% for training and 20% for validation. We used mean-squared error as a metrics of the reconstruction error of the autoencoder. Training continued until the reconstruction error converged (final error: ~3.0e-04). When reconstructing the validation data with the trained autoencoder, we set the threshold for classifying abnormal performance data as the mean reconstruction error +2 standard deviations. We regarded the performance data with reconstruction errors exceeding the threshold as abnormal performance data. The proportion of validation data classified as normal values was defined as the specificity of the classifier. The proportion of patient data classified as abnormal values was defined as the sensitivity of the anomaly classifier (Figure 2).

### Measurement of biomechanical function

Although Musician’s dystonia is a disorder in which dexterity is impaired primarily during the performance of specific tasks, it is known that there are differences in hand perceptive or sensory-motor functions unrelated to specific motor tasks, such as longer temporal discrimination threshold (TDT), compared to healthy individuals (18, 19). Conversely, it has been reported that TDT is not altered in musicians with dystonia (20). Furthermore, biomechanical factors seem to be related to musicians with dystonia, at least in a subgroup (11, 21). Therefore, in addition to the piano performance experiments, we also examined the biomechanical functions of the fingers that are not directly related to the performance, which include the maximum strength of each finger, the reduction rate of force exertion during simultaneous force exertion with all fingers, the independence, agility, moving range in extension/flexion and abduction/adduction rotation directions of each finger and the temporal sensory ability.

For assessing finger strength, simultaneous force exertion, independence, and agility, we used a custom-made force sensor device designed to measure the force exerted by the five fingers. Participants put each of the five fingers on different independent force sensors (TAL220, SparkFun), the location of which can be customized so as to fit with the hand of the individual participants. The wrist and elbow were immobilized during the measurement, and only the fingers were allowed to exert force for flexion. In measuring the maximum finger strength, participants were instructed to press the force sensor as strongly as possible with each finger individually. In measuring simultaneous force exertion, participants were instructed to press the force sensor with all fingers simultaneously. The reduction rate of force exertion was calculated as the ratio of finger force during simultaneous force exertion and the maximum finger strength of each finger (22, 23). During the assessment of finger movement independence, participants were instructed to press the force sensor at 20% of full strength with only the designated finger for 5 s with all fingers on the force sensor. The independence was calculated as the 1 – the ratio of the designated finger force and the sum of the other fingers’ force (24). To assess movement agility, participants were instructed to tap the force sensor repetitively with the designated finger at the fastest rate for 5 s (Supplementary Figure 1-1).

The measurement of the finger moving range was conducted with keeping the wrist and the upper arm fixed to the base. The range of motion was measured by the relative values of the posture sensor that was attached to the proximal phalangeal bone and back of the hand. The posture sensor, consisting of an inertial measurement unit (ICM-20948, TDK InvenSense), calculates the three-dimensional posture by utilizing the Earth’s gravity and magnetic field (Supplementary Figure 1-2).

The participants in this study included right-handed and left-handed individuals. Furthermore, the dystonia patients did have different patterns of dystonic movements and were affected either on their left or right hand. Therefore, we focused on the asymmetry between the left and right hands. The asymmetricity is defined as


$$\mathrm{Assym}=\|\begin{array}{l} {\left[z_{L,\mathrm{thumb}},\;z_{L,\mathrm{index}},\;z_{L,\mathrm{middle}},\;z_{L,\mathrm{ring}},\;z_{L,\mathrm{pinky}}\right]}^{T}-\\ {\left[z_{R,\mathrm{thumb}},\;z_{R,\mathrm{index}},\;z_{R,\mathrm{middle}},\;z_{R,\mathrm{ring}},\;z_{R,\mathrm{pinky}}\right]}^{T} \end{array}\|$$

where z indicates the z-score of each biomechanical function score, the first subscript indicates the hand (L: left and R: right), and the second subscript indicates the finger.

We used the Temporal Order Judgment Threshold (TOJT) to assess the temporal sensory ability of participants, as the commonly used TDT is susceptible to judgment bias. TOJT was measured using a custom-made tactile stimulator consisting of a microcontroller (STM32 Nucleo-32, STMicroelectronics) and two solenoids (ZH0-0420S-05A4.5, Shenzhen Zonhen Electric Appliances). Participants placed their right index and middle fingertips on the solenoids. Two tactile stimuli were sequentially delivered to the fingers in a randomized order by the two solenoids with a short time interval. Subsequently, the participants were required to answer which finger received the tactile stimuli first. The time interval between two stimuli was shortened if the response was correct and increased if incorrect according to the ZEST method in psychophysics (25). TOJT of the participants was defined as the time interval at which the correction rate exceeded 75% (Supplementary Figure 1-3). We opted for mechanical stimulation instead of electrical stimulation for simplicity, while adhering to the target body parts of previous studies that measure TDT of patients with MD (20). Prior to measurements, we ensured that the magnitude of the mechanical stimuli was sufficiently above each participant’s tactile threshold.

We adopted different approaches for each of the video footage and biomechanical function datasets because we prioritized simplicity. The main purpose of this study is to propose simple diagnostic support methods for MD, targeting physicians with no expertise in music performance. We aimed to provide straightforward hand biomechanical function tests to support the diagnosis of MD. Therefore, instead of employing a machine learning approach that discriminates MD based on the distribution of diverse test scores, we employed conventional statistical methods to identify tests where significant differences emerge. Furthermore, integrating video footage and hand biomechanics data into a single machine-learning algorithm was challenging due to the different modalities of the datasets.

## Results

### Video-based classification of Musician’s dystonia

**Figure 3:**
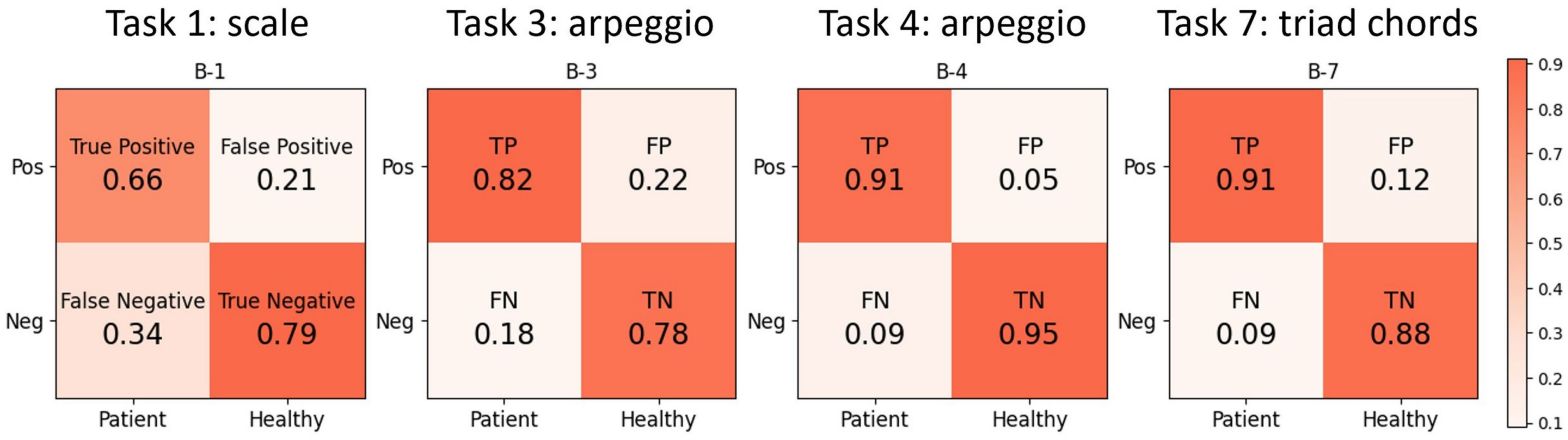
The result of the video-based classification of MD using the autoencoder. The autoencoder is trained to reconstruct the hand kinematics of healthy pianists, thus it cannot reconstruct the hand kinematics of pianists with MD. We set the threshold for abnormal performance data as the mean reconstruction error +2 standard deviations when reconstructing the validation data of healthy pianists. The sensitivity of the autoencoder exceeds the chance level for 4 tasks and exceeds 90% in arpeggio task and triad chords striking task.

We evaluated the sensitivity and specificity of the anomaly classifier using the autoencoder trained with the kinematic data of healthy pianists during the performances for each pianistic task. For four out of the 11 tasks, including a scale, arpeggios, and thirds chord striking (tasks 1, 3, 4, and 7), the sensitivities were higher than the chance level (>50%). The sensitivity and specificity were 66 and 79% for task 1 (scale), 82 and 78% for task 3 (arpeggio), 91 and 95% for task 4 (arpeggio with black keys), and 91 and 88% for the for task 7 (thirds chord striking), respectively. These results indicate the highest classification accuracy for pianistic task 4, which involved striking the black and white keys by opening and closing the hand with the thumb-under maneuver. For the remaining seven tasks, the sensitivity of the autoencoder was below the chance level (<50%), which indicates a failure of classification between patients and controls with these tasks (Figure 3).

### Assessment of biomechanical functions

**Figure 4:**
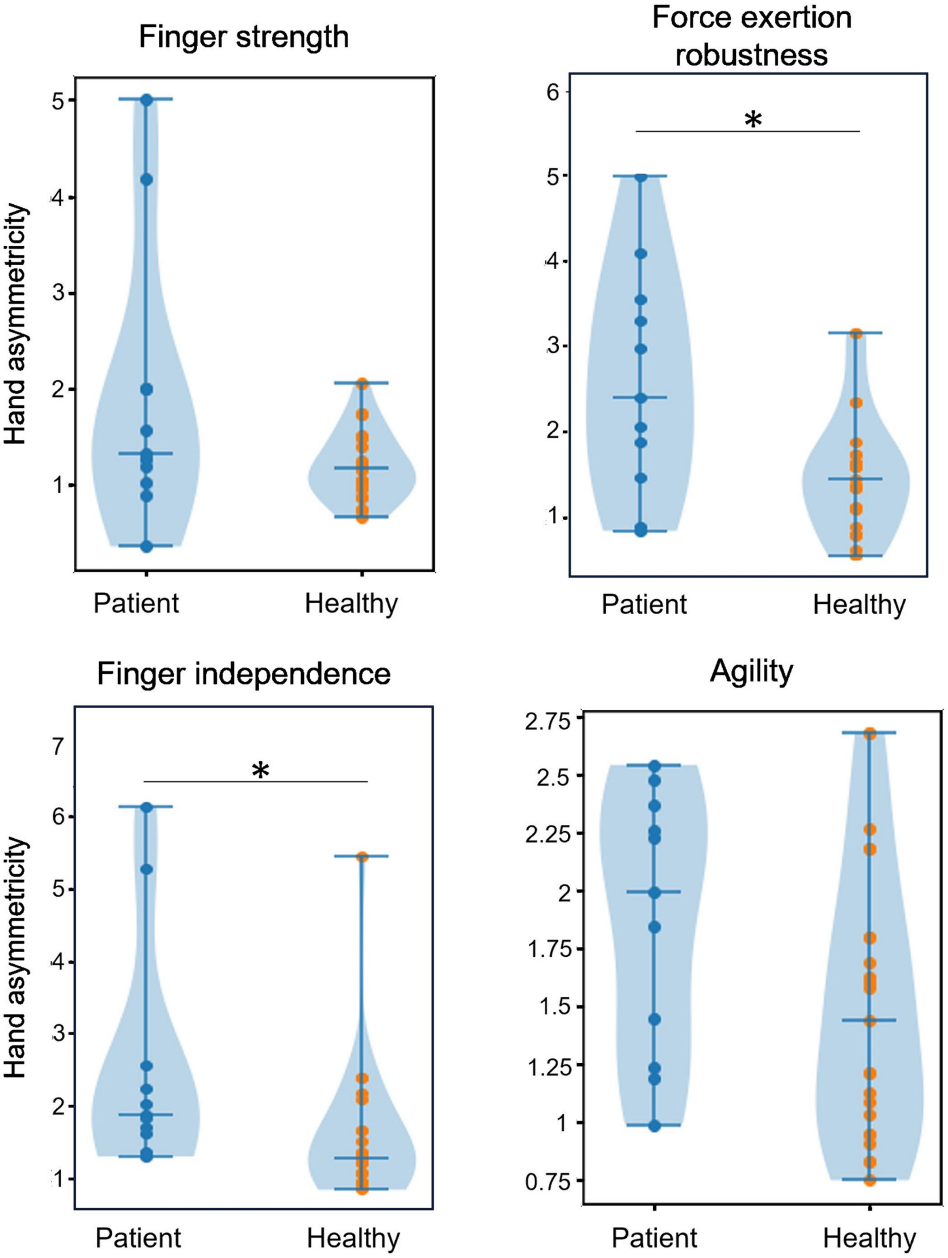
The result of the hand asymmetricity of biomechanical function measurement. There were significant asymmetricity differences between the pianists with MD and healthy pianists at the robustness of simultaneous force exertion and finger independence, which require simultaneous control of multiple fingers. On the other hand, we did not find any significant differences on the hand asymmetricity of strength and agility of individual fingers.

We evaluated the asymmetricity of the biomechanical functions between the left and right hands. There were no group-wise significant differences in the asymmetricity of strength of each finger, agility, and flexibility between the left and right hands between healthy pianists and pianists with MD. By contrast, asymmetricity in the force exertion with all fingers and finger independence were larger in the pianists with MD (Man-Whittney U test with Benjamini-Hochberg correction for multiple comparisons: *p* = 0.017, and *p* = 0.013). We found no significant differences in the asymmetricity of the flexibility between healthy pianists and pianists with MD in any direction. We did not observe significant differences in the TOJT between healthy pianists and pianists with MD nor between the right and left hands of pianists with MD (Figure 4).

## Discussion

In this study, we proposed a machine-learning algorithm capable of detecting focal dystonia in pianists with an accuracy of approximately 90% by using anomaly detection and a commercially available video camera. The diagnosis of MD has been readily prone to misdiagnosis due to the task-specific manifestation of symptoms and the need for specialized knowledge on complex movement coordination in musical performance. Despite using only video images during the performance, the method proposed in this study achieved high accuracy in the classification of movements between pianists with and without MD. Furthermore, by assessing the finger biomechanical functions, which are unspecific to musical performance, the accuracy of the classification was improved.

Several methods using scale performance and specific tasks on the piano keyboard have been proposed to objectively assess the nature and degree of MD (13). However, these methods require an electronic piano and a non-commercially available program based on data from MIDI technology, which is difficult to handle and requires time-consuming post-processing. This makes them difficult to use in non-specialized institutions in musician’s medicine.

An innovative aspect of the method in this study is the use of simple video footage, allowing for the development of an automatic diagnostic assistance system using a smartphone camera. Furthermore, this study used an anomaly detection method using an autoencoder based on unsupervised learning rather than supervised learning. Unsupervised learning is appropriate here because the prevalence of musician’s dystonia is estimated to be around 1% (1). Therefore, collecting performance data from healthy pianists is much easier than collecting data from dystonia patients. In general, creating a high-performance machine learning model requires a large amount of high-quality training data. In the case of supervised learning, normal and abnormal labels need to be annotated to the performance data to teach the model how to classify. However, when there is a significant imbalance in the number of labels, the features of the minority labels tend to be ignored, such as in the case of rare disorders, which makes it difficult to improve accuracy even by increasing the number of training data (26). By contrast, in the case of anomaly detection methods using unsupervised learning, learning the features of normal data enables the segregation between the performances of pianists with focal dystonia and healthy pianists. Therefore, further accuracy improvement is possible by increasing the training data of healthy pianists. Moreover, by expanding the dataset of healthy pianists, the algorithm can address the diagnosis of an early stage of MD, in which its symptoms only appear on limited passages, and provide follow-up assessments where the pattern of dystonic movements changes with treatment.

The differences in MD detection accuracy among the tasks may offer insights for improving the algorithm. The kinematic features of each task likely account for these differences. For example, tasks requiring minimal finger movement, such as repetitive chord striking (B-9), may not adequately elicit symptomatic movement, which might complicate MD detection. Additionally, considerable inter-individual differences within the training data from healthy pianists could affect the autoencoder’s ability to calculate abnormalities for certain tasks. To enhance the algorithm’s accuracy, carefully selecting performance tasks for training data for the autoencoder and incorporating the domain knowledge of experienced physicians would be beneficial.

The proposed algorithm facilitates the diagnosis of musicians’ dystonia for neurologists and movement disorders specialists who are not experienced in analyzing evenness or sound quality in pianists. More importantly, we now have an easily applicable objective measure for follow-up studies, such as assessing treatment effects. This has been proven to be time-consuming and laborious (27). Plucked instruments can be the next target for extending this algorithm to the diagnosis of MD on other instruments. Video footage of the right hand can be applicable for assessing hyperflexion and hyperextension in a similar manner as proposed in this study. The algorithm for plucked instruments can be extendable for the left hand of string players and in woodwind instruments according to the same principles. Although a single camera was utilized in this study for simplicity, this setup may limit the accuracy of kinematic measurements in less kinematically constrained tasks such as drumming, violin bowing, and guitar playing. This issue could be addressed by altering the source of the kinematic data. Employing conventional motion capture with a machine-learning algorithm appears promising (28). In addition, a whole-body estimation algorithm using multiple RGB or depth cameras is a relatively simple and promising method for such low-constrained tasks. Recently, methods for detecting facial, finger, and whole-body feature points using machine learning have been developed, enabling almost real-time feature point detection (15, 29, 30). Therefore, this method is potentially applicable to the diagnosis of focal dystonia targeting effectors other than fingers, such as embouchure and feet. In principle, the proposed algorithm could potentially be adapted to diagnosing other movement disorders by creating specific patient datasets.

Furthermore, the proposed algorithm can be applied to the treatment of MD. Since it quantifies the degree of abnormality as the autoencoder’s reconstruction error, the algorithm can be directly used for quantitative evaluation of the treatment effect. In addition, this algorithm can quantitatively evaluate the similarity of kinematics, as the performance kinematics are represented by feature vectors in the middle layer of the autoencoder. Therefore, it can assist in establishing treatment plans by analyzing patient data with similar kinematics to determine the most effective treatments applied in comparable cases. An intriguing observation was abnormal asymmetry of the force exertion of all fingers and finger independence between the left and right hand in dystonia patients. These biomechanical functions are not specific to the instrumental performance that triggers symptoms of MD. However, the former is related to simultaneous control of multiple fingers, which is required for musical performance (e.g., playing a chord), whereas the latter is related to a task requiring inhibitory control between adjacent fingers (31). Abnormal surround inhibition of adjacent fingers at resting state in pianists with MD has been reported in neurophysiological studies using transcranial magnetic stimulation (32). The present behavioral task of evaluating finger independence has a clinical advantage over transcranial magnetic stimulation in that the abnormal inhibitory control of the fingers can be readily evaluated. Significant differences between healthy pianists and patients with focal dystonia were evident in motor functions requiring multi-finger coordination, which is unnecessary in daily manual tasks (33). It is unclear whether the multi-finger coordination function was impaired due to focal dystonia or whether the innately unskilled multi-finger coordination function became a factor in the development of focal dystonia. However, abnormalities of generalized, not task-specific, biomechanical dysfunctions associated with task-specific dystonia have been described by Wilson (11) and Leijnse (34). These results emphasize the urgent need for longitudinal assessment of these functions to prevent the development of Musician’s dystonia.

This study has potential limitations. The MD and healthy control groups were not well-matched in terms of gender and age. The demographics of most MD group participants (elderly males with extensive piano training experience) made recruiting a better-matched healthy control group difficult. However, we believe that the kinematic and video parameters in these extremely overlearned and automated tasks in healthy, professional, fully trained pianists are independent of gender and do not alter until senescence (which we excluded). Indeed, we have demonstrated remarkable stability of expert pianists in kinematic and acoustic parameters in scale-playing skills in earlier longitudinal studies (35). Furthermore, Gründahl et al. showed that the severity of symptoms is not age-dependent using video-rating conducted by expert pianists, indicating that the age of the pianists with MD does not affect their movement patterns (4). Additionally, the algorithm currently only discriminates the presence of symptoms and does not classify their severity. Future work will involve establishing the criteria of severity that combine audio criteria and the abnormality of finger kinematics. Creating a large performance dataset of audio and video recordings from a wide range of pianists, including both healthy individuals and patients across various ages and genders, would be beneficial for developing a machine learning-based diagnostic system for MD and its early detection. Furthermore, the proposed algorithm does not provide the reason of the classification because of the lower explainability of the CNN architecture. Other non-parametric classification techniques, such as anomaly detection algorithms using tree structures like the Isolation Tree, may be potential candidates offering better explainability. However, tree-structured algorithms require feature engineering for classification. The variability in the dystonic patterns of the participants, as presented in Table 1, made designing appropriate feature variables challenging. To address the issue of the lower explainability of CNN-based algorithms, techniques for visualizing the basis of image classification in CNN networks, such as Grad-CAM (36), may enhance the explainability of our anomaly detection algorithm. Although to identify the best classifier is out of scope of the present study, it can be significant to apply such classification techniques in future studies.

## Data availability statement

The raw data supporting the conclusions of this article will be made available by the authors, without undue reservation.

## Ethics statement

The studies involving humans were approved by Institutional Review Board at Leibniz University Hannover. The studies were conducted in accordance with the local legislation and institutional requirements. The participants provided their written informed consent to participate in this study.

## Author contributions

TO: Conceptualization, Data curation, Formal analysis, Investigation, Methodology, Project administration, Software, Validation, Visualization, Writing – original draft, Writing – review & editing. SF: Conceptualization, Formal analysis, Funding acquisition, Investigation, Methodology, Project administration, Supervision, Validation, Writing – original draft, Writing – review & editing. AL: Conceptualization, Investigation, Methodology, Validation, Writing – original draft, Writing – review & editing. EA: Conceptualization, Formal analysis, Funding acquisition, Investigation, Methodology, Project administration, Resources, Supervision, Validation, Writing – original draft, Writing – review & editing.

## Supplementary material

Supplementary FIGURE S1 Schematic illustration of the hand biomechanical function 591 measurement. Finger strength, reduction rate of force exertion during simultaneous force 592 exertion with all fingers, agility, and independence were measured using a custom-made 593 force sensor. Moving range in extension/flexion direction and abduction/adduction of each 594 digit are measured using a posture sensor consists of an accelerometer and a magnetic field 595 sensor. Time discrimination threshold was measured using a custom-made tactile 596 stimulator consists of two solenoids. Two solenoids sequentially stimulate index and middle 597 finger of the participant with short time interval and the participant answer which finger 598 perceived tactile stimuli first. TOJT was defined as the time interval at which the correction 599 rate exceeded 75%.
