# Supplementary figures and images for: Video-based diagnosis support system for pianists with Musician’s dystonia
Source: Front Neurol. 2024 Jul 2;15:1409962. doi: 10.3389/fneur.2024.1409962 (PMC11250081; doi:10.3389/fneur.2024.1409962)

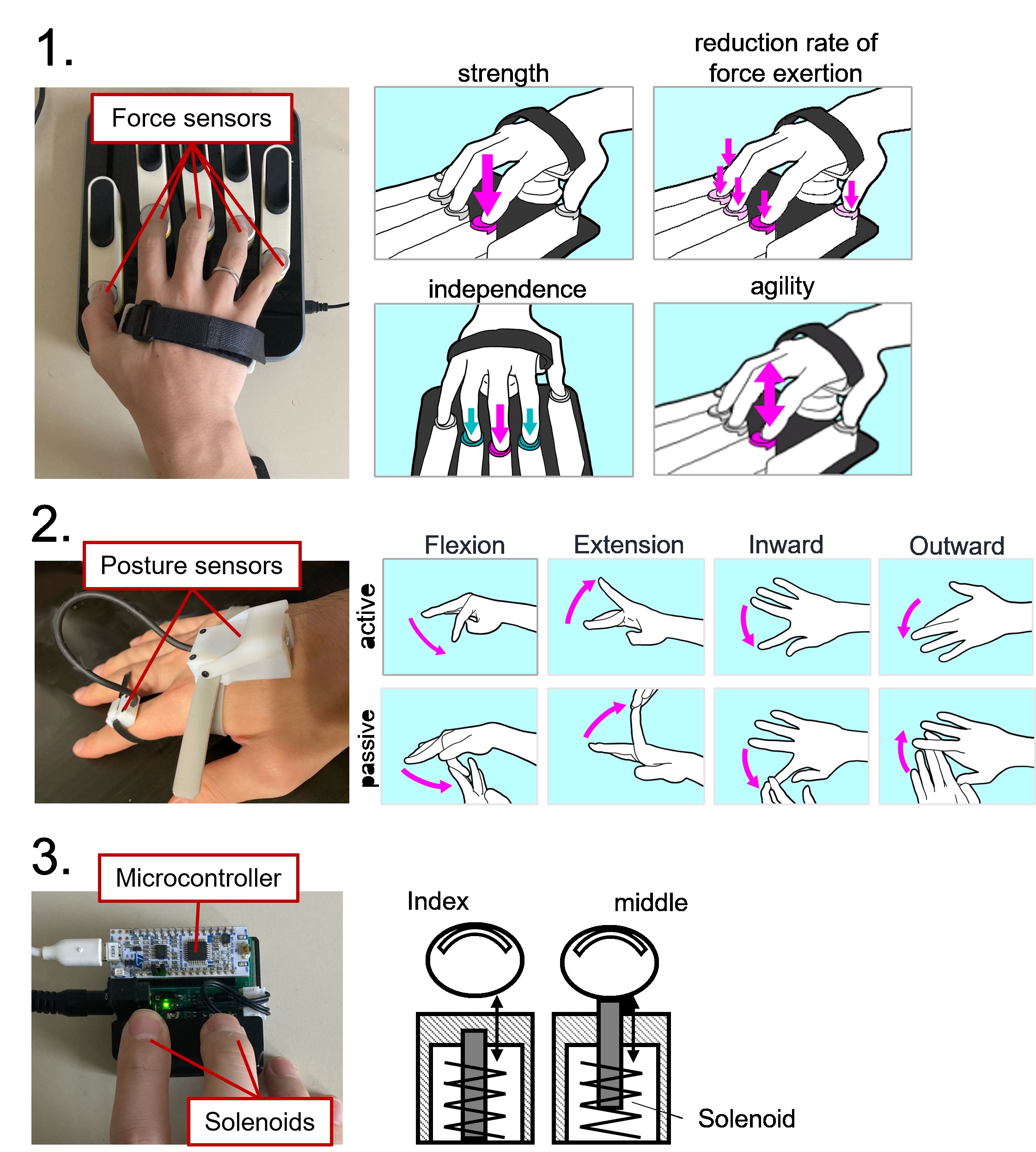

Supplement: Supplementary FIGURE S1 — Schematic illustration of the hand biomechanical function 591 measurement. Finger strength, reduction rate of force exertion during simultaneous force 592 exertion with all fingers, agility, and independence were measured using a custom-made 593 force sensor. Moving range in extension/flexion direction and abduction/adduction of each 594 digit are measured using a posture sensor consists of an accelerometer and a magnetic field 595 sensor. Time discrimination threshold was measured using a custom-made tactile 596 stimulator consists of two solenoids. Two solenoids sequentially stimulate index and middle 597 finger of the participant with short time interval and the participant answer which finger 598 perceived tactile stimuli first. TOJT was defined as the time interval at which the correction 599 rate exceeded 75%. [file Image_1.JPEG]
